# Bilateral MR-Guided Focused Ultrasound Pallidothalamic Tractotomy for Parkinson's Disease With 1-Year Follow-Up

**DOI:** 10.3389/fneur.2021.601153

**Published:** 2021-02-09

**Authors:** Marc N. Gallay, David Moser, Anouk E. Magara, Fabio Haufler, Daniel Jeanmonod

**Affiliations:** ^1^SoniModul, Center for Ultrasound Functional Neurosurgery, Solothurn, Switzerland; ^2^Praxisgemeinschaft für Neurologie, Bern, Switzerland; ^3^ETH Zürich, Department of Management, Technology, and Economics, Zurich, Switzerland

**Keywords:** high-intensity MR-guided focused ultrasound, pallidothalamic tractotomy, Parkinson's disease, incisionless, bilateral, parkinson

## Abstract

**Objective:** Bilateral stereotactic neurosurgery for advanced Parkinson's disease (PD) has a long history beginning in the late 1940s. In view of improved lesioning accuracy and reduced bleeding risk and in spite of long-standing caveats about bilateral approaches, there is a need to investigate bilateral MR-guided focused ultrasound (MRgFUS) interventions. We hereby present the clinical results of bilateral pallidothalamic tractotomy (PTT), i.e., targeting of pallidal efferent fibers below the thalamus at the level of Forel's field H1, followed for 1 year after operation of the second side.

**Methods:** Ten patients suffering from chronic and therapy-resistant PD having received bilateral PTT were followed for 1 year after operation of the second side. The primary endpoints included the Unified Parkinson's Disease Rating Scale (UPDRS) scores in on- and off-medication states, dyskinesias, dystonia, sleep disturbances, pain, reduction in drug intake, and assessment by the patient of her/his global symptom relief as well as tremor control.

**Results:** The time frame between baseline UPDRS score and 1 year after the second side was 36 ± 15 months. The total UPDRS score off-medication at 1 year after the second PTT was reduced by 52% compared to that at baseline on-medication (*p* < 0.007). Percentage reductions of the mean scores comparing 1 year off- with baseline on-medication examinations were 91% for tremor (*p* = 0.006), 67% for distal rigidity (*p* = 0.006), and 54% for distal hypobradykinesia (*p* = 0.01). Gait and postural instability were globally unchanged to baseline (13% improvement of the mean, *p* = 0.67, and 5.3% mean reduction, *p* = 0.83). Speech difficulties, namely, hypophonia, tachyphemia, and initiation of speech, were increased by 58% (*p* = 0.06). Dyskinesias were suppressed in four over four, dystonia in four over five, and sleep disorders in three over four patients. There was 89% pain reduction. Mean L-Dopa intake was reduced from 690 ± 250 to 110 ± 190.

**Conclusions:** Our results suggest an efficiency of bilateral PTT in controlling tremor, distal rigidity, distal hypobradykinesia, dyskinesias, dystonia, and pain when compared to best medical treatment at baseline. Larger series are of course needed.

## Introduction

Bilateral stereotactic neurosurgery for Parkinson's disease (PD) has a long history, beginning in the late 1940s. Bilateral approaches, staged or concomitant, have been mostly published in the 1960s (1–6), in the pre-L-DOPA era. Clinical results, although lacking modern criteria for reporting, were nevertheless impressive in the context of limited imaging modalities for guidance to reach deeply located brain targets. These results were probably favored by a patient collective who certainly did not match our current, older, and therapy-resistant one. Radiofrequency (RF) thermocoagulation of the posteroventral internal (or medial) pallidum was re-investigated following the seminal work of Laitinen in the early 1990s (7). Case series of bilateral pallidotomies followed between the 1990s and early 2000s (8–16) until the deep brain stimulation (DBS) technique came to dominate the field.

Our current approach, i.e., targeting the pallidal efferent fibers in the subthalamus, was proposed by Meyers (17) and later developed by others (18, 19). It was re-actualized by Jeanmonod and co-workers first with RF (20, 21) and since 2011 with MR-guided focused ultrasound (MRgFUS) (22, 23). It was proposed to target specifically the pallidothalamic tract just below the thalamus at the level of Forel's field H1, where the ansa lenticularis and the fasciculus lenticularis converge (24). This intervention was named pallidothalamic tractotomy (PTT), a more precise denomination than campotomy or subthalamotomy as Forel described three fiber fields in the subthalamus (25). PTT corresponds functionally to an optimized pallidotomy because it allows, with limited tissue ablation (around one third), an extensive liberation of the thalamocortical dynamics from pallidal over-inhibition while leaving the thalamus intact and simultaneously reducing the risk of capsular or optic nerve encroachment. A prospective open-label study and a case report describing similar subthalamic approaches with RF have been published recently (26, 27).

MRgFUS was shown to be a safe and accurate lesioning technique (28, 29). The clinical efficacy and side effects of the MRgFUS PTT will therefore be mainly related to the chosen target. It is common knowledge that PD is a bilateral disease in the vast majority of advanced and therapy-resistant cases. Despite all efforts of the neurological and functional neurosurgical community, a large number of patients are reluctant to undergo DBS surgery even when meeting the criteria for referral to a DBS center (30). In this context, in view of improved lesioning accuracy, reduced bleeding risk, and suppression of infectious risk and in spite of the long-standing caveats about bilateral approaches, there is a need to investigate bilateral MRgFUS interventions (31). We hereby present the clinical results of bilateral PTT applied in 10 patients followed for 1 year after their second PTT.

## Methods

### Study Context

This is a single-center, retrospective observational analysis of a series of patients suffering from chronic and therapy-resistant PD who received staged or contemporaneous bilateral PTT under normal standard of care. Only patients with a follow-up of 1 year after operation of the second hemisphere were included in the analysis. All the patients included in this analysis provided informed consent to the surgical intervention.

### Ethics

All patients treated in this retrospective analysis signed an informed consent form after having been fully informed about the treatment, its results, and its risks. They gave their written permission to publish their anonymized data. No additional ethical approval was sought because MRgFUS PTT has been approved by the Federal Once of Public Health of Switzerland and monitored since 2015 in the context of a registry controlled by this office.

### Selection Criteria

The selection criteria for MRgFUS PTT were described previously (22). Only mixed and tremor-dominant PD forms were considered in this study. Patients were referred to our specialized center in order to be treated with MRgFUS. Bilateral treatment was considered for patients having benefited from unilateral PTT and meeting the same criteria for the second body side, i.e., (1) idiopathic PD, (2) at least 1 year of therapy resistance, characterized by insufficient efficacy of L-Dopa, with symptom control during <50% of the day in on-state, gastrointestinal, or other side effects, fluctuations (on-off phenomena), and on-medication dyskinesias (choreoathetosis), (3) strong (3/4 on a scale of 0–4 over 4) intensity of symptoms, (4) strongly diminished quality of life (3/4), and (5) Montreal Cognitive Assessment (MoCA) score higher or equal to 20/30. The time interval between both PTTs was not prefixed but was rarely below 1 year. Contemporaneous bilateral PTT was exceptionally considered in patients displaying intact thalamocortical reserves (i.e., absence of brain atrophy and normal cognitive status).

Regardless of the presence or absence of an indication for DBS surgery, the patients in this series chose, prior to the preoperative evaluation in our center, not to receive DBS for personal reasons in spite of the ubiquitous standard of care recognition for DBS.

### Procedure

All procedures were performed in a 3T MR imaging system (GE Discovery 750, GE Healthcare, Milwaukee, WI, USA) using the ExAblate Neuro device (InSightec, Haifa, Israel). A standardized bilateral PTT was performed according to previously published protocol (22, 32) either concomitantly or in two sessions. For each target, five to seven sub-units of 1.5 × 1.5 × 3.0 mm were placed. Sonications were applied with the shortest possible duration and corresponding power in order to provide for each target lesion sub-unit a thermal dose of at least 240 cumulative equivalent minutes (CEM). For patients who received unilateral PTT prior to 2017, retreatment of the first PTT to increase the lesion size was performed during the operative session for the second side. An example of such retreatment was published recently (33). Target reconstruction and determination of lesioning accuracy were performed for all patients (34).

### Clinical Evaluation and Outcome Measures

The primary endpoints, measured before the second PTT and 1 year after it, both compared with baseline examination, included (1) the Unified Parkinson's Disease Rating Scale (UPDRS) scores (35) in on- and off-medication state, (2) dyskinesias (choreo-athetosis), (3) off- and on-dystonia, (4) sleep disturbances (UPDRS-score, item 41), (5) pain (UPDRS-score, item 17), (6) reduction in drug intake, and (7) assessment by the patient of her/his global symptom relief and tremor control for the entire body. On-medication examinations were performed 1–2 h after the intake of a dosage of L-Dopa according to the patient's usual best response. The secondary endpoints included (1) MoCA (36) performed at baseline, 2 days after the first side, and before, 2 days, and 1 year after the second side, (2) the questionnaire for the activities of daily living according to Bain et al. (37), (3) the World Health Organization Quality of Live (WHOQOL bref), and (4) the Hospital Anxiety and Depression Scale (HADS) (38). On- and off-medication UPDRS examinations were performed at baseline and before the second PTT. At 1 year after the second side, only off-medication UPDRS was recorded (seven over 10 patients having stopped L-Dopa intake).

If not specified otherwise, values given in the text are means ± standard deviation.

### Statistics

Statistical analysis of quantitative scores compared with baseline was carried out with non-parametric Wilcoxon matched-pairs signed-ranks test (StataCorp. 2013. Stata Statistical Software: Release 13. College Station, TX: StataCorp LP). Statistically significant *p*-values based on non-parametric Wilcoxon matched-pairs signed-ranks test were marked with an asterisk (^*^). The calculated effects are based on the comparison of the pre-intervention baseline on-medication with the 1-year off-medication follow-up. Thus, results are relying on the assumption of an unchanged severity of the patient's on-medication test scores over the whole study period. Due to the small sample size, the statistical analysis has to be interpreted with caution.

## Results

Patient characteristics are summarized in Table 1. Over 11 patients meeting the inclusion criteria described in “METHODS,” one of them was lost for postoperative follow-up because of a frail general state of health and long-distance travel. Nine patients had a tremor-dominant and one had a mixed PD form. On-medication examinations were performed after the intake of 100–200 mg L-Dopa. One patient responded at very low (50 mg) L-Dopa dosage. Off-medication assessments were performed 12 h after the last L-Dopa intake in 6/10 and between 4 and 12 h in 4/10 patients. All the patients benefited at least for their second PTT from our optimized targeting protocol (32, 33). PTT was performed staged in eight patients and contemporaneous in two. The latter two patients had no hemispheric atrophy and normal cognition. They were, in terms of parkinsonian symptomatology, severely affected, and their personal situations presented humanitarian dimensions. The time frame between baseline UPDRS score and 1 year after the second side was 36 ± 15 months (median: 33 months, range: 14–59). The interval between both PTTs was 20 ± 10 months (median: 16.5, range: 5–38). Four patients received a retreatment of their first PTT to increase the lesion size during the second PTT session. High targeting accuracy, analyzed in previous studies (29, 34), prevented encroachment on the internal capsule or mammillothalamic tract in all 10 patients.

**Table 1:** Characteristics of 10 patients with chronic therapy-resistant Parkinson's disease.

Patients	10
Bilateral pallidothalamic tractotomy (PTT): stage/one session	8/2
Time frame between first Unified Parkinson's Disease Rating Scale (UPDRS) and 1 year postoperative UPDRS after second side: mean ± SD, median (months)	36 ± 15, 33 (14–59)
Symptom duration [mean ± SD (min; max), median] (years)	10.2 ± 4.6 (7; 23), 9
Time interval between both PTTs [mean ± SD (min; max), median] (months)	20 ± 10 (5; 38), 16.5
Mean age at first treatment (years)	63 ± 5
Females	5/10
Ethnicity	9 Caucasians, 1 Indo-aryan
Preoperative mean modified Hoehn and Yahr stage (39) (min; max)	2.7 (1;4)
Mean modified Hoehn and Yahr stage (min; max) before second side	2.5 (2;3)
Retreatment of first PTT to increase lesion size and contralateral PTT in one session	4/10

**Figure 1:**
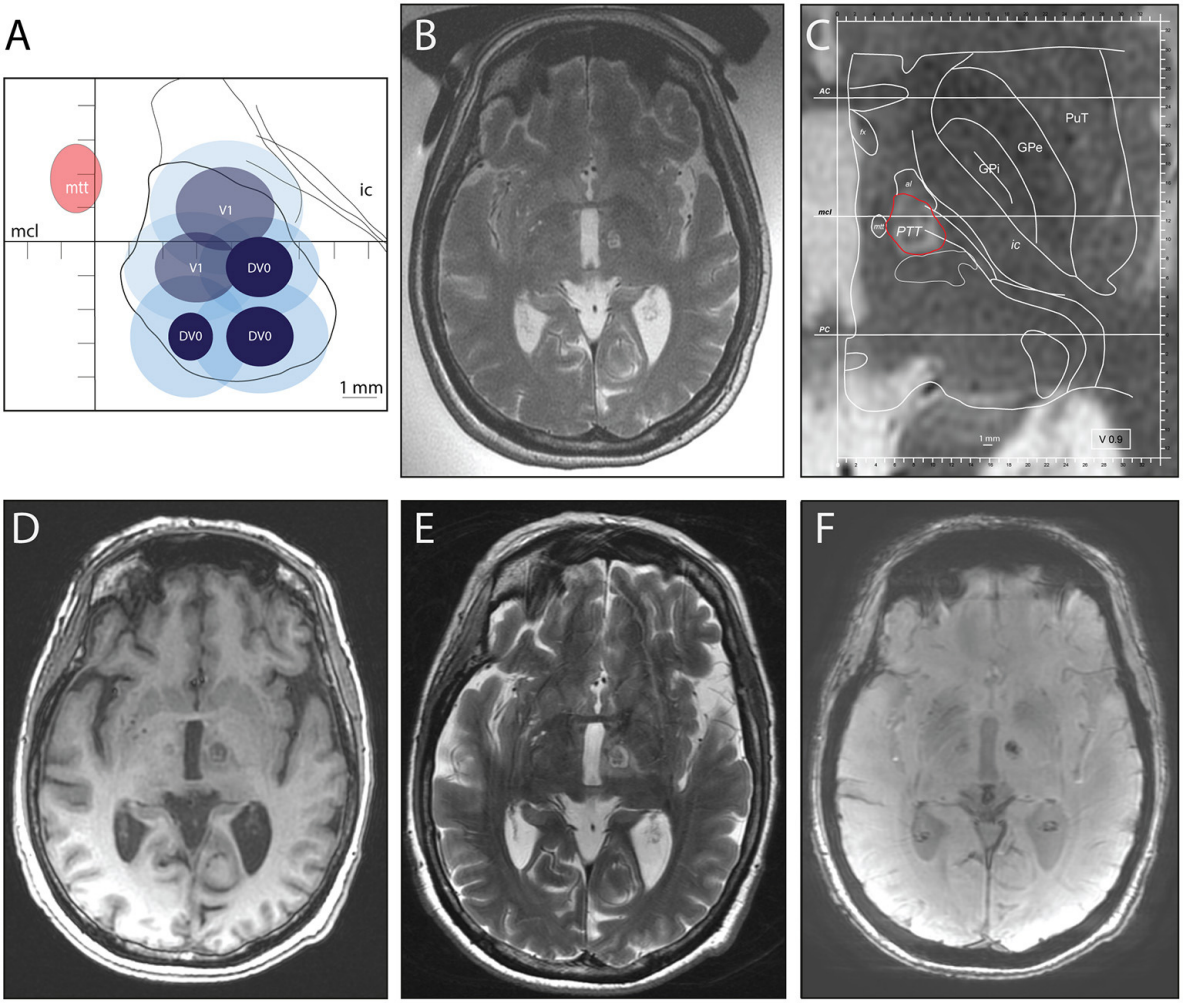
Second side (left hemisphere) pallidothalamic tractotomy (PTT) performed 17 months after right-sided PTT. **(A)** There were 18 cumulative equivalent minutes (CEM; light blue) and 240 CEM (dark blue) thermal dose surfaces projected on a standardized PTT targeting map [modified from Gallay et al. (32)]. Target sub-units were realized either at the intercommissural plane (DV0) or 1 mm below (V1). **(B)** Intraoperative MR axial T2 image of the performed target (with the body coil and transducer filled with water). **(C)** Enlarged intraoperative MR T2 axial image with projected modified version of plan DV0 of Morel's Atlas (22). In red, outline of the planned PTT target. At 2 days after PTT: MR axial T1 image **(D)**, T2 image **(E)**, and susceptibility weighted angiography image. The first right-side PTT is visible in **(D, F)**.

The skull density ratio was 0.63 ± 0.1 (range: 0.45–0.76). Sonication power and energy applied to reach final temperatures were 938 ± 280 (W) (range: 600–1,350) and 11,130 ± 5,380 (J) (range: 5,900–22,800), respectively. The lesion volume calculated on MR T2 images 2 days after PTT was 290 ± 120 mm^3^. Figure 1 illustrates the thermal doses applied and MR images of the lesion at the end of a second side PTT and 2 days after it. The CEM at 43°C thermal dose surfaces, calculated by the ExAblate Software (Insightec, Ltd.), were plotted on a modified map of the PTT target (33).

### Primary Outcome Measures

**Figure 2:**
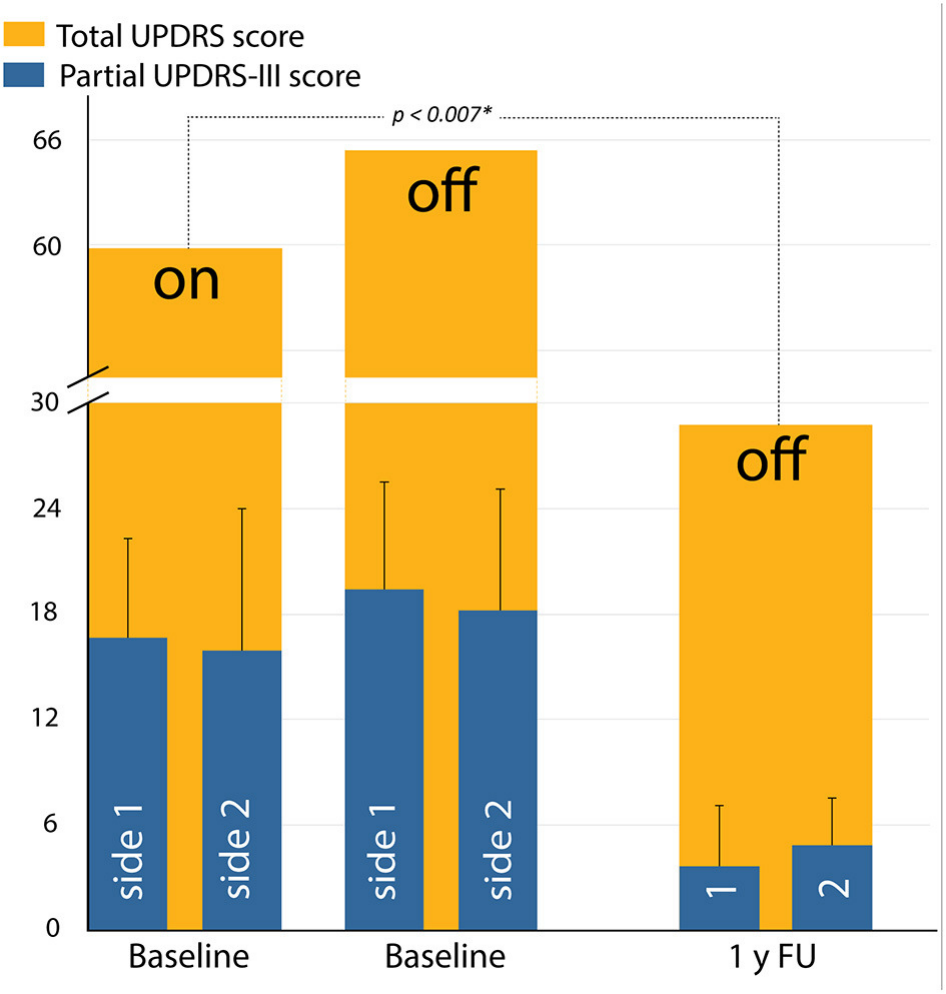
Average scores showing total Unified Parkinson's Disease Rating Scale (UPDRS) scores (orange) in on- and off-medicated state, measured preoperatively (baseline) and at 1-year follow-up. Average scores with standard deviation shown in blue are the evolution of partial UPDRS III scores of the first (labeled side 1 or 1) and the second (labeled side 2 or 2) PTTs. *, statistically significant.

**Figure 3:**
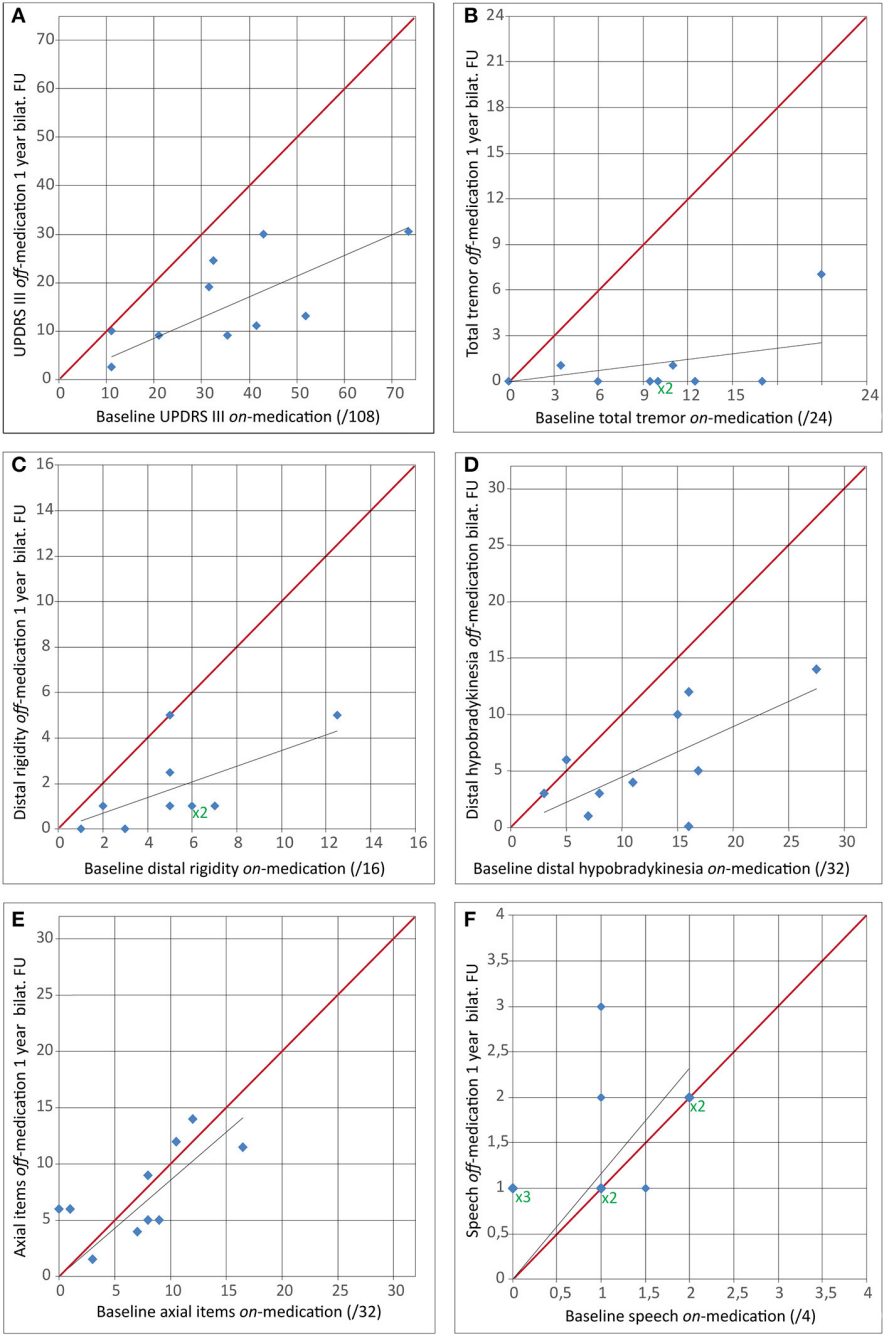
Comparison of baseline on-medication score with 1-year follow-up after second side off-medication for **(A)** Unified Parkinson's Disease Rating Scale (UPDRS) III score, **(B)** total tremor (UPDRS items 20 and 21), **(C)** distal rigidity (UPDRS items 22.2–5.), **(D)** distal hypobradykinesia (UPDRS items 23–26), **(E)** axial items (UPDRS items 18, 19, 22, 27, and 28–31), and **(F)** speech (UPDRS item 18). For superimposed dots, X2 or X3 (green) multipliers were added. The red line indicates an unchanged measured score from the baseline on-medication to the 1-year follow-up off-medication.

UPDRS scores and selected separated UPDRS items are presented in Tables 2, 3, Figures 2, 3. The total UPDRS score off-medication 1 year after the second PTT was 29 ± 11. It corresponded to a 52% reduction as compared to baseline on-medication examination (*p* < 0.007^*^). Figure 2 shows total UPDRS and partial UPDRS III scores after the second PTT. The total UPDRS or UPDRS III scores were both not improved before the second PTT compared to baseline even with a 60% (6.6 ± 6.1 vs. 17 ± 6, *p* = 0.025^*^) reduction of the mean of the partial UPDRS III score for the first operated side on-medication and 66% in off-medication state (6.7 ± 6.2 vs. 20 ± 6, *p* = 0.017^*^). In contrast and counterbalancing these improvements, the UPDRS III score for the second operated side showed a global increase of its mean by 71% (*p* = 0.025^*^) in on- and 50% (*p* = 0.036^*^) in off-medication state during the mean interval period of 20 months between both examinations. UPDRS I and UPDRS II changed from baseline to last follow-up from 3.7 ± 2.4 to 2.2 ± 1.7 (*p* = 0.13) and from 15 ± 6 to 10 ± 3 (*p* = 0.07), respectively. Figure 2 and Table 2 show the evolution of partial UPDRS III scores from baseline on- and off-medication examinations, before the second side, and at 1-year follow-up after the second side. Figure 3 depicts the evolution of tremor, distal rigidity, distal hypobradykinesia, axial items (including gait and postural instability), and speech from on-medication baseline examination to 1-year follow-up off-medication after bilateral PTT.

**Table 2:** Changes from baseline, before the second pallidothalamic tractotomy (PTT), and at 1 year after bilateral PTT.

	**Baseline**	**Before second side**	***p* (Wilcoxon)**	**1 year after second**	***p* (Wilcoxon)**
	**(*n* = 10)**	**(*n* = 8)**		**side (*n* = 10)**	
Total Unified Parkinson's Disease Rating Scale (UPDRS), on-medication (/199)	60 ± 23	54 ± 16	0.89		
Total UPDRS, off-medication (/199)	65 ± 25	57 ± 14	0.48	29 ± 11	0.007^*^
UPDRS I (/16)	3.7 ± 2.4	3.4 ± 1.6	0.82	2.2 ± 1.7	0.13
UPDRS II (/52)	15 ± 6	15 ± 4	0.72	10 ± 3	0.07
Speech (item 5) (/4)	0.9 ± 0.9	1.6 ± 0.8	0.053^*^	2.1 ± 0.7	0.02^*^
UPDRS III on-medication (/108)	35 ± 18	30 ± 14	0.83		
UPDRS III off-medication (/108)	41 ± 20	33 ± 12	0.4	16 ± 9	0.005^*^
First operated side, UPDRS III, on-medication (/36)^a^	17 ± 6	6.6 ± 6.1	0.025^*^		
First operated side, UPDRS III, off-medication (/36)^a^	20 ± 6	6.7 ± 6.2	0.017^*^	3.6 ± 3.5	0.005^*^
Second operated side, UPDRS III, on-medication (/36)^a^ (*n* = 8)	9.3 ± 9.1	16 ± 8	0.025^*^		
Second operated side, UPDRS III, off-medication (/36)^a^ (*n* = 8)	12 ± 11	18 ± 7	0.036^*^	4.9 ± 2.7	0.09
Tremor, both sides on-medication (/24)^b^	11 ± 6	6.7 ± 4.8	0.53		
Tremor, both sides off-medication(/24)^b^	13 ± 6	7.5 ± 3.9	0.08	0.9 ± 2.1	0.006^*^
Tremor, first operated side on-medication (/12)^c^	6.0 ± 3.2	1.7 ± 2.6	0.065		
Tremor, first operated side off-medication (/12)^c^	7.5 ± 2.7	1.4 ± 1.9	0.014^*^	0.4 ± 0.9	0.008^*^
Tremor, second operated side on-medication (/12)^c^	3.5 ± 3.3	5.0 ± 3.2	0.44		
Tremor, second operated side off-medication (/12)^c^	4.6 ± 3.8	6.1 ± 3.0	0.57	0.5 ± 1.2	0.03^*^
Rigidity, both sides on-medication (/16)^d^	5.3 ± 3.2	3.9 ± 3.0	0.83		
Rigidity, both sides off-medication (/16)^c^	6.4 ± 3.8	5.4 ± 2.4	0.44	1.8 ± 1.8	0.006^*^
Distal hypobradykinesia both sides on-medication (/32)^e^	12.6 ± 6.9	7.9 ± 6.5	0.44		
Distal hypobradykinesia both sides off-medication (/32)^e^	14.0 ± 7.7	11.9 ± 5.1	0.78	5.8 ± 4.5	0.01^*^
Axial items UDPRS III on-medication (/32)^f^	7.5 ± 5.1	6.0 ± 4.3	1.00		
Axial items UDPRS III off-medication (/32)^f^	8.0 ± 5.2	8.6 ± 4.3	0.62	7.4 ± 4.0	0.88
Speech on-medication (/4) (item 18)	1.0 ± 0.8	0.7 ± 0.8	0.93		
Speech off-medication (/4) (item 18)	1.0 ± 0.8	1.1 ± 0.8	0.32	1.5 ± 0.7	0.06
Gait (item 29) on-medication (/4)	0.8 ± 0.7	1.0 ± 0.9	0.4		
Gait (item 29) off-medication (/4)	1.0 ± 0.6	1.0 ± 0.7	1.00	0.7 ± 0.6	0.67
Postural instability (item 30) on-medication (/4)	1.0 ± 1.0	0.8 ± 0.5	0.94		
Postural instability (item 30) off-medication (/4)	1.0 ± 1.0	1.1 ± 0.6	0.94	0.9 ± 0.7	0.83

*For UPDRS scores, higher values indicate stronger impairments. P-values were calculated with baseline using non-parametric Wilcoxon matched-pairs signed-ranks test. In the last column, p-values are given for comparisons between postoperative off-medication and baseline on-medication (except UPDRS I and II items)*.

n, number of interventions included in the analysis;

*, *p-values which reached statistical significance after correction*.

a *UPDRS III items 20.2, 20.4, 21.1, 22.2, 22.4, 23.1, 24.1, 25.1, 26.1 or 20.3, 20.5, 21.2, 22.3, 22.5, 23.2, 24.2, 25.2, and 26.2 on operated side (i.e., partial UPDRS III)*.

b *UPDRS III items 20 and 21*.

c *UPDRS III items 20.2, 20.4, 21.1 or 20.3, 20.5, and 21.2*.

d *UPDRS III items 22.2, 22.3., 22.4, and 22.5*.

e *UPDRS III items 23, 24, 25, and 26*.

f *UPDRS III items 18, 19, 22, 27, 28, 29, 30, and 31*.

**Table 3:** At 1 year after bilateral pallidothalamic tractotomy (PTT) off- vs. baseline on-medication examination.

	**Percentage reduction of the mean at 1 year after bilateral PTT off- vs. baseline on-medication**	***p* (Wilcoxon)**
Total tremor^a^	91% (0.9 ± 2.1 vs. 11 ± 6)	0.006^*^
Total distal rigidity^b^	67% (1.8 ± 1.8 vs. 5.3 ± 3.2)	0.006^*^
Total distal hypobradykinesia^c^	54% (5.8 ± 4.5 vs. 12.6 ± 6.9)	0.01^*^
Axial items^d^	1.3% (7.4 ± 4.0 vs. 7.5 ± 5.1)	0.88
Postural instability^e^	5.3% (0.9 ± 0.7 vs. 1.0 ± 1.0)	0.83
Gait^f^	13% (0.7 ± 0.6 vs. 0.8 ± 0.7)	0.67
Speech^g^	−58% (1.5 ± 0.7 vs. 1.0 ± 0.8)	0.06

*P-values given after non-parametric Wilcoxon matched-pairs signed-ranks test. n = 10*.

*, *values which reached statistical significance*.

a *UPDRS III items 20 (extremities only) and 21*.

b *UPDRS III items 22.2–5*.

c *UPDRS III items 23–26*.

d *UPDRS III items 18, 19, 22, 27, 28, 29, 30, and 31*.

e *Unified Parkinson's Disease Rating Scale (UPDRS) III item 30*.

f *UPDRS III item 29*.

g *UPDRS III item 18*.

Mean tremor reduction, calculated based on the UPDRS at 1 year after the second side off-medication compared to baseline UPDRS on-medication, was 93% (range: 67–100, median: 100%). It represented a 91% percentage reduction of the mean total tremor (*p* = 0.006^*^). The percentage reduction of mean distal rigidity and distal hypobradykinesia was 67% (*p* = 0.006^*^) and 54% (*p* = 0.01^*^), respectively. Axial items were globally unchanged to baseline (1.3% improvement of the mean, *p* = 0.88). Gait and postural instability were improved, but not significantly (13% improvement of the mean, *p* = 0.67, and 5.3% mean reduction, *p* = 0.83). For example, as to gait, at 1 year, four patients had improved their gait, three patients scored worse, and three were unchanged. No change in gait scores exceeded 1 point in the UPDRS.

At 1 year follow-up after bilateral PTT, dyskinesias (choreoathetosis) were suppressed in four over four, dystonia in four over five, and sleep disorders in three over four patients. Pain was suppressed in four over seven and strongly reduced in three. Mean UPDRS item 17, quantifying pain, was reduced from 2.6 ± 1.3 to 0.3 ± 0.5 (89% reduction). L-Dopa intake was 690 ± 250 at baseline and 110 ± 190 at 1 year after the second PTT. Seven patients had stopped any drug intake at that time, and all patients had stopped their dopamine agonists. The three other patients had an L-Dopa intake of 200, 300, and 600 mg. For these three patients, no on-medication examinations were performed at 1 year as they reported no significant differences between their on and off times. In addition, the mean value of their UPDRS-III off-medication examination was 32 ± 14 compared to 27 ± 9 in the seven other patients (L-Dopa free), indicating a low risk of a relevant effect of L-Dopa due to insufficient wash-out.

Difficulties for speech were increased by 58% (mean 1.5 ± 0.7 vs. 1.0 ± 0.8) in the postoperative 1-year off-medication vs. baseline on-medication comparison (*p* = 0.06). 2 days after first PTT (*n* = 8), speech was improved in three, worsened in one, and unchanged in four patients. When checked at 2 days after the second PTT (*n* = 10), speech was improved in two, worsened in one, and unchanged in seven compared to baseline. At 1-year follow-up, speech was improved in one, worsened in five, and unchanged in four patients. The recorded speech difficulties were hypophonia, tachyphemia, and initiation of speech, but not dysarthria. At 1 year after the second PTT, seven patients out of 10 had speech therapy.

### Subjective Assessments

According to patient's subjective assessments, global symptom relief for both body sides was rated as 82 ± 25% (*n* = 10, median: 95%, range: 20–100), and subjective percentage of tremor relief for the whole body was 95 ± 4% (*n* = 9, median: 95%, range: 90–100) at 1 year after bilateral PTT. Subjective speech difficulties (UPDRS item 5) at 1 year after the second side were increased in eight patients, reduced in one, and unchanged in one as compared to baseline (mean 2.1 ± 0.7 vs. 0.9 ± 0.9, *p* = 0.018). At 2 days after the second PTT, they were only increased in one, reduced in one another, and unchanged in eight.

Sialorrhea was an issue in five patients at baseline and was present in six patients (two newly developed, one normalized) at 1-year follow-up. Two patients had improved their swallowing issues present at baseline, but three others developed new mild to moderate difficulties between baseline and the last follow-up.

### Secondary Outcome Measures

MoCA mean scores did not change from baseline (28 ± 2, range: 26–30), before the second PTT (28 ± 2, range: 25–30), and at 1 year after bilateral PTT (28 ± 3, range: 20–30). At 1 year after bilateral PTT, two patients scored higher and seven were unchanged, but one had a lower score by six points compared to baseline. This worsening was recorded at 2-day postoperative examination (−3 points) and 1 year after his second PTT (−3 points). This patient received an accurately placed lesion without encroachment on adjacent structures. HADS (13 ± 3 baseline *vs* 11 ± 5 at 1 year after bilateral PTT). The World Health Organization Quality of Life questionnaire (WHOQOL bref) (93 ± 11 vs. 95 ± 11) and Activities of Daily Living questionnaire (45 ± 10 vs. 42 ± 15) mean values were all improved, but these changes were not statistically significant.

### Adverse Events

Sonications were painful for a few seconds in one patient. There was no bleeding, no infection, no ballism, and no paralysis. There was no acute state of confusion, no suicidal ideations, and no development of psychiatric symptoms. All patients could be discharged from the clinic 1 day after PTT. One patient suffered from a hiccup as well as difficulties in breathing and speech which lasted over months but were regressive at 10 months. One patient fell twice on the first postoperative day after his second PTT, without injury, and reported to be slightly dragged to his right side. His gait improved over the first postoperative weeks and was normalized and even improved to baseline at 3 months after bilateral PTT. At 1 year after bilateral PTT, one patient experienced episodes of uncontrollable laughter. Additionally, he reported blepharospasms mostly during emotionally challenging moments.

## Discussion

This is a small series of 10 bilateral PTT interventions for PD with MRgFUS. PTTs were performed according to our latest targeting protocol developed to optimize target coverage and taking into account interindividual variability and using preplanned focal point displacements, short sonication durations, and thermal dose control (32, 33). To justify brain surgery and particularly in a context of lesioning procedure, an intervention should provide globally better results than drug therapy. Hence, as therapy resistance was a requirement to indicate surgery, it appeared natural to compare the results at follow-up in an off-medication state with their on-medication baseline. Only thus could the superior relief obtained by surgery be assessed as compared to best medical treatment. We cannot exclude the relevance of an insufficient medication wash-out (see “RESULTS”) for the three patients still taking L-Dopa postoperatively.

At 1 year after bilateral PTT, the patients reported a high global symptom relief for both body sides, in line with objective measurements. The addition of patient reports on their own surgical outcomes (10, 22, 40), which cover their whole daily living time, provides a useful addition to the clinical examination, which was obviously limited in time.

Axial symptoms were the most difficult to control. The mean scoring for axial items, gait, and postural instability remained statistically unchanged at 1 year after bilateral PTT. Our data confirm an observation which has been well-recognized since years: there is a gradient of efficiency in postoperative akinetic symptom control, with distal (extremity or appendicular) localization benefitting from the highest level of relief and axial (midline) symptoms being more resistant to surgical treatment (41). Objective scoring revealed 58% worsening of speech (that is hypophonia, tachyphemia, and reduced speech initiation) at 1 year off-medication examination compared to on-medication baseline. Due to the size of the cohort, small intensity of worsening, and presence of improvement in one patient (*p* = 0.06), this value was not statistically significant but suggests a strong trend. Reporting on difficulties for speech highlights the obvious limitation of being recorded on the UPDRS scoring system on a 0 to 4 scale. As pointed out by Parkin and coworkers (13), “sensitivity to change is reduced even further if most patient scores are in the lower range. These factors give rise to a statistical floor effect, whereby if many scores are at the bottom of the range, any resampling error will tend to increase the average as these scores can only change in one direction.”

Chronic stimulation of the subthalamic nucleus has also been shown to cause speech worsening in up to 69% of patients (42). A recent review analyzed the occurrence of dysarthria in bilateral thalamotomies (31). We attribute the fact that our speech difficulties did not qualify for dysarthria to the sparing of the thalamus in the context of a subthalamic approach. The targeting accuracy provided by the MRgFUS technique allows one to strongly reduce the risk of a corticobulbar syndrome due to capsular involvement, a situation which did not happen in our patients.

As gait, postural stability, and other axial functions did not significantly improve, surgery, as it has been well-recognized since years before (2, 43), should be offered very restrictively to patients complaining dominantly about symptoms affecting these dimensions. The issue of speech and of speech therapy has to be addressed with patients and relatives prior to bilateral PTT, and we are implementing a better pre- and postoperative assessment of speech through intensified collaborations with speech therapists and the introduction of the Voice Handicap Index questionnaire (44).

What could be the reason of the described lack of improvement of axial symptoms and the worsening of speech? An insufficient coverage of the PTT target does not seem to be a good explanation in view of the guidance provided by detailed intra-operative data, leading us to complement four targets. Such a partial therapeutic effect is in any case not easily compatible with the fact that it is always the axial akinetic symptoms which resist. At this point, a relevant piece of evidence from this study must be considered: at 2 days after their second PTT, on examination and subjectively, only one patient displayed a worsening of his voice as compared to an objective and subjective speech worsening in five and eight patients at 1 year after the second PTT, respectively. This difference indicates a mechanism which has progressed along time. In harmony with the current concept of neurodegeneration, the possibility of disease progression unimpeded by the surgical act has to be considered. We speculate about an alternative explanation. The cingulate motor areas are, among others, a good candidate to fulfill the role of a crosstalk platform for the integration of psychomotor functions (45). Examples abound of the sometimes overwhelming effects of emotions on motricity, e.g., voice modulation and the freezing of gait. An outspoken placebo effect has been described in parkinsonian patients (46). Gait is the key to independence and voice to communication, two essential psychosocial components of human life. The strength of the neurodegeneration concept imposed on PD patients and threatening their bodily and mental functions cannot be underrated. It can explain the modest improvements of emotionality and quality of life described by our patients in spite of very significant and multiple symptom reductions.

The mean MoCA score was unchanged between baseline and at 1 year after bilateral PTT despite a reduction of six points in one patient. In a prospective study from Denmark (47), the risk of dementia in Parkinson's disease over a period of 4 years was found to be 95.3 per 1,000 person-years in a PD population with shorter symptom duration. According to these data, two to three MoCA reductions could have happened regardless of interventions.

The results obtained after bilateral pallidotomies were reviewed by Counihan and Cosgrove (8). Three case series of bilateral pallidotomies with more than 30 patients (11, 13, 14) confirmed the sustained improvements of distal motor functions as well as near total abolition of L-Dopa-induced dyskinesias. Interestingly, concerns about cognitive impairments after pallidotomies have been raised mostly in very small series (<10 patients), but not in larger ones (8, 11, 13, 16). The surgical experience may have played a role in this discrepancy, allowing to avoid encroachment on the internal capsule by increased accuracy and/or smaller lesion volumes.

The small cohort size of this analysis and its retrospective *post hoc* characteristics represent significant limitations. The large size of the reported effects may serve, however, as a valuable basis for further studies.

## Conclusion

Our results, in spite of the cohort size limitation, suggest an efficiency of bilateral PTT in controlling tremor, distal rigidity, distal hypobradykinesia, dyskinesias, dystonia, and pain compared to best medical treatment at baseline over a mean period of 36 months. The axial symptoms were unchanged, and speech got worse over the year after the second PTT. Larger series are of course needed.

## Data Availability Statement

The raw data supporting the conclusions of this article will be made available by the authors, without undue reservation.

## Ethics Statement

Ethical approval was not provided for this study on human participants because all patients treated with this protocol signed an informed consent form after having been fully informed about the treatment, its results and risks. No additional ethical approval was sought because MRgFUS PTT has been approved by the Federal Once of Public Health (FOPH) of Switzerland and all patients were part of a Registry controlled by this office. The patients/participants provided their written informed consent to participate in this study.

## Author Contributions

MG: conception and design of the study, data acquisition and analysis, interpretation of the data, and co-drafted the manuscript. DJ: conception and design, data acquisition, interpretation of the data, and co-drafted the manuscript. DM, AM, and FH: co-drafted the manuscript. FH and MG: statistical analysis. All authors read and approved the final manuscript.

## Conflict of Interest

MG, DM, AM, and DJ were employed by SoniModul, Ltd., Center for Ultrasound Functional Neurosurgery, Solothurn, Switzerland. The Center for Ultrasound Functional Neurosurgery in Solothurn and its employees did not receive any financial support by any medical company, including Insightec, Ltd, for the whole time of this study. The remaining author declares that the research was conducted in the absence of any commercial or financial relationships that could be construed as a potential conflict of interest.
